# Diffuse Large B-cell Lymphoma Presenting as a Hypermetabolic Mesenteric Root Mass Without Preoperative Imaging Suspicion of Lymphoma: A Case Report

**DOI:** 10.7759/cureus.108988

**Published:** 2026-05-16

**Authors:** Jinglei Li, Quan Jiang, Weiqi Lu

**Affiliations:** 1 Department of Retroperitoneal Soft Tissue Surgical Oncology, Zhongshan Hospital, Shanghai, CHN

**Keywords:** diffuse large b-cell lymphoma, fdg pet/ct, frozen section, intra-abdominal lymphoma, mesenteric root mass, surgical biopsy

## Abstract

Diffuse large B-cell lymphoma (DLBCL) may occasionally present as an intra-abdominal mass, but a dominant mesenteric root presentation can closely mimic other aggressive abdominal malignancies. Markedly elevated fluorodeoxyglucose (FDG) uptake on positron emission tomography/computed tomography (PET/CT) suggests high tumor metabolic activity but is not histologically specific.

A 72-year-old male presented with a two-week history of abdominal pain and was found to have a retroperitoneal mass on imaging. On physical examination, a hard, ill-defined, mobile mass was palpable above the umbilicus on deep palpation, without tenderness. Contrast-enhanced CT and FDG PET/CT demonstrated a dominant soft-tissue mass closely associated with adjacent bowel loops and mesenteric vascular structures. PET/CT showed intense FDG uptake, with a maximum standardized uptake value (SUVmax) of 35.5 on standard imaging and approximately 40.0 on delayed imaging. Preoperative imaging favored an aggressive abdominal malignancy rather than lymphoma. Because percutaneous biopsy was considered high risk due to the lesion’s location, the patient underwent surgical exploration. Intraoperative frozen-section analysis suggested a hematolymphoid malignancy, prompting the surgical team to avoid extensive bowel or mesenteric vascular resection and to limit the procedure to diagnostic tissue acquisition. Final pathology confirmed DLBCL, non-germinal center B-cell type, with a high proliferative index.

This case highlights that very high FDG uptake in an abdominal mass should be interpreted as evidence of aggressive tumor biology rather than as a marker of a specific histologic diagnosis. When lymphoma is not suspected preoperatively and image-guided biopsy is unsafe, intraoperative frozen-section assessment can be critical in redirecting surgical management and avoiding non-therapeutic radical resection.

## Introduction

Diffuse large B-cell lymphoma (DLBCL) is the most common subtype of non-Hodgkin lymphoma and is biologically heterogeneous, with variable nodal and extranodal presentations [1-3]. Current classification requires integration of morphology, immunophenotype, cell-of-origin features, and selected molecular or cytogenetic findings, because these data affect diagnostic precision and may influence prognostic interpretation [1-4]. Although systemic immunochemotherapy remains the cornerstone of treatment for most patients with DLBCL, adequate tissue sampling is essential before treatment initiation to distinguish DLBCL from high-grade B-cell lymphoma, indolent lymphoma with transformation, carcinoma, sarcoma, gastrointestinal stromal tumor (GIST), and other mimics [5-7]. In abdominal presentations, especially those involving the mesenteric root, obtaining tissue can be technically difficult because lesions may be surrounded by bowel loops and major mesenteric vessels.

Fluorodeoxyglucose (FDG) positron emission tomography/computed tomography (PET/CT) is valuable for staging and response assessment in FDG-avid lymphomas and has been incorporated into modern lymphoma staging and response criteria [8-10]. DLBCL is typically FDG-avid, and high uptake may correlate with aggressive histology or proliferative biology [11,12]. However, intense FDG uptake is not specific for lymphoma. High-risk GIST, high-grade soft tissue sarcoma, poorly differentiated carcinoma, neuroendocrine carcinoma, melanoma, and metastatic disease may all present as hypermetabolic intra-abdominal tumors [13-16]. Therefore, a very high maximum standardized uptake value (SUVmax) should be interpreted as a marker of aggressive biology rather than a definitive indicator of histologic subtype.

To our knowledge, no prior case report has specifically addressed the combined scenario of a hypermetabolic mesenteric root mass not preoperatively suspected to be lymphoma, unsafe percutaneous access, and intraoperative frozen-section analysis redirecting the operative plan from radical resection to diagnostic biopsy. This report aims to illustrate how frozen-section evaluation can prevent non-therapeutic extensive surgery when preoperative tissue diagnosis is not feasible.

## Case presentation

A 72-year-old male presented with a two-week history of abdominal pain and was found to have a retroperitoneal mass on imaging one week before admission. He had initially sought medical attention at an outpatient clinic in late March 2026, where a contrast-enhanced CT of the abdomen revealed a mass at the mesenteric root. He was subsequently referred to our institution and admitted in April 2026.

His medical history was notable for hypertension of 10 years' duration, well controlled with antihypertensive medication. He had a remote history of schistosomiasis in childhood. He denied fever, night sweats, or unintentional weight loss.

On physical examination, a hard, ill-defined, mobile mass was palpable above the umbilicus on deep palpation, without tenderness or rebound tenderness. No peripheral lymphadenopathy was detected. The remainder of the examination was unremarkable.

Laboratory investigations, including complete blood count, serum biochemistry (liver and renal function, lactate dehydrogenase), and coagulation profile, showed no significant abnormalities. Serum lactate dehydrogenase was within the normal range.

Contrast-enhanced CT of the abdomen with small bowel protocol demonstrated a heterogeneous-density soft-tissue mass at the mesenteric root, measuring approximately 70 × 66 mm, with mild enhancement and ill-defined margins (Figure 1). Branches of the superior mesenteric artery and superior mesenteric vein coursed through the lesion. No enlarged retroperitoneal or pelvic lymph nodes were identified. Incidental findings included gallbladder sludge, a small splenic enhancing focus (approximately 7 mm), and bilateral renal cysts. The radiological impression was a malignant tumor of the mesenteric root with involvement of adjacent mesenteric vessels.

**Figure 1:**
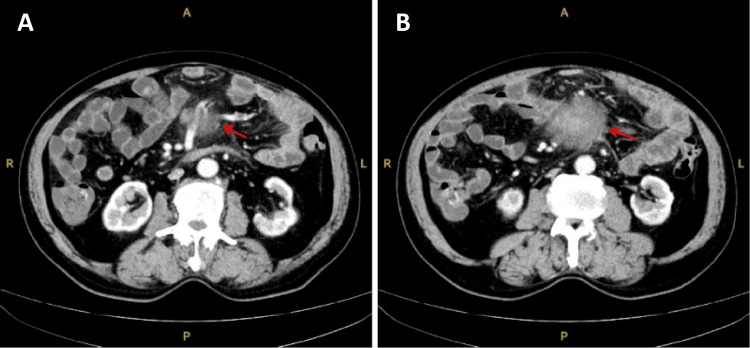
Contrast-enhanced CT showing the mesenteric root mass. (A) Tumor invasion of mesenteric vascular branches (red arrow). (B) Tumor invasion of an adjacent bowel loop at a different level.

FDG PET/CT performed in April 2026 showed a hypermetabolic soft-tissue mass in the retroperitoneal/mesenteric root region. The lesion demonstrated markedly increased FDG uptake, with an SUVmax of 35.5 and delayed uptake of approximately 40.0. The report described suspected malignant tumor involvement of surrounding vessels and bowel, with possible adjacent peritoneal involvement or metastasis. Mild FDG uptake was also noted in bilateral submandibular, parapharyngeal, left axillary, and bilateral inguinal lymph nodes, but these findings did not lead to a preoperative impression of lymphoma. Additional findings included pulmonary inflammatory changes, a right renal cyst, and prostatic hyperplasia with calcification (Figure 2).

**Figure 2:**
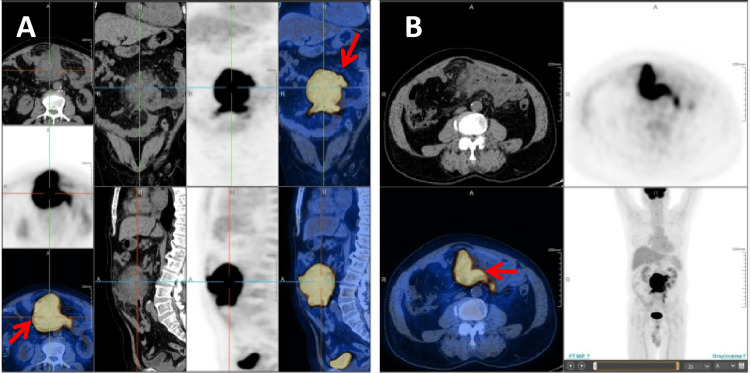
Fluorodeoxyglucose (FDG) PET/CT demonstrating intense metabolic activity within the mesenteric root mass. (A) Intense hypermetabolic activity (red arrow) in the primary mass invading adjacent mesenteric vessels and bowel loop. (B) A small satellite lesion near the primary tumor with elevated metabolic activity.

Based on the clinical presentation, physical examination, and all preoperative imaging findings, the preoperative differential diagnosis included primary mesenteric sarcoma, GIST, metastatic disease, and lymphoma; however, lymphoma was considered less likely given the dominant mass pattern without generalized lymphadenopathy.

Percutaneous biopsy was considered technically hazardous due to the deep mesenteric root location, surrounding bowel loops, and adjacent vascular structures. After multidisciplinary discussion, including interventional radiology consultation, the consensus was that surgical exploration with tissue diagnosis was the safest diagnostic approach. The preoperative surgical plan accounted for the possibility of a resectable aggressive solid tumor, with possible extensive bowel resection and mesenteric vascular division if necessary. At laparotomy, the mass was found to involve adjacent small bowel and mesenteric vascular structures, with small surrounding nodules. The gross appearance raised the possibility of lymphoma. Tissue was obtained for intraoperative frozen-section analysis, which suggested a hematolymphoid malignancy. The operative team discussed the findings with the patient's family, explaining that lymphoma would typically be managed with systemic therapy rather than extensive resection. With the family's agreement, the operation was limited to tissue diagnosis, and extensive bowel and mesenteric vascular resection was not performed (Table 1).

**Table 1:** Timeline of diagnostic evaluation and key management decisions. FDG: fluorodeoxyglucose; SUVmax: maximum standardized uptake value.

Diagnostic domain	Key findings
Clinical-radiologic presentation	Dominant mesenteric root mass surrounded by small bowel loops and closely associated with mesenteric vessels; preoperative imaging favored aggressive non-lymphomatous intra-abdominal malignancy.
PET/CT	Marked FDG uptake; SUVmax of 35.5 and delayed uptake of approximately 40.0.
Biopsy feasibility	Percutaneous biopsy was considered high risk after multidisciplinary and interventional radiology evaluation.
Intraoperative finding	Mass involving adjacent bowel and mesenteric vessels, with small surrounding nodules.
Frozen section	Hematolymphoid malignancy suspected, prompting abandonment of planned extensive resection.
Final diagnosis	Diffuse large B-cell lymphoma, non-germinal center B-cell type.

The patient recovered uneventfully after surgery and was referred for hematology evaluation and systemic therapy planning. At the time of manuscript preparation, long-term follow-up and treatment response data were not yet available.

Pathologic findings

Gross examination of the mesenteric tumor showed a gray-yellow mass measuring approximately 3.6 × 2.2 × 1.5 cm. Histologic examination revealed a poorly differentiated malignant tumor with diffuse infiltrative growth (Figure 3).

**Figure 3:**
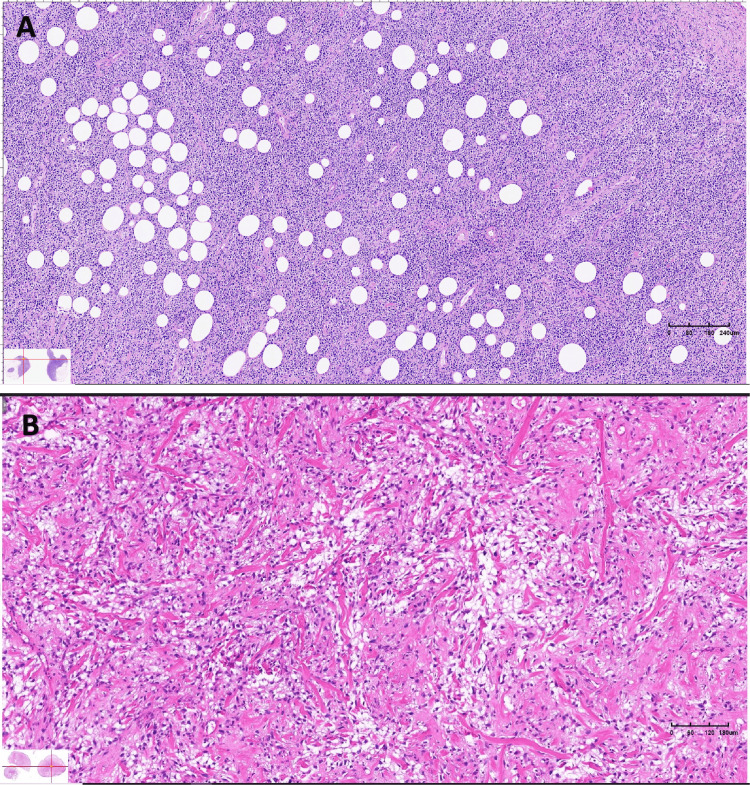
Histopathologic features of the mesenteric mass. (A) H&E staining (40×) showing diffuse infiltration by sheets of medium to large atypical lymphoid cells with effacement of normal architecture. (B) Additional H&E field showing high-grade lymphoproliferation with brisk mitotic activity. Immunophenotyping and molecular findings are summarized in Supplementary Tables A1-A4.

Immunohistochemistry showed that the tumor cells were strongly positive for CD20 and positive for CD21 and BCL6, with MUM1 positivity. The expression of CD10, BCL2, CD30, CD5, CD23, Cyclin D1, SOX11, ALK, EBER, CD56, and CD43 was negative. CD3 highlighted only scattered background T cells (see Supplementary Table A1 for the full immunophenotypic results and staining intensity/proportion). The Ki-67 proliferation index was approximately 90%, MYC protein expression was approximately 40%, and p53 showed abnormal strong positivity in approximately 90% of tumor cells. BRG1 and INI-1 expression was retained, and the mismatch repair proteins MLH1, MSH2, MSH6, and PMS2 were retained (see Supplementary Table A1).

Fluorescence in situ hybridization (FISH) demonstrated BCL6 rearrangement, while MYC and BCL2 rearrangements were not detected. IRF4 rearrangement was negative, and MYD88 (exon 5) mutation was not detected (see Supplementary Tables A2-A4 for detailed molecular/FISH results). Gene rearrangement testing supported clonal lymphoid proliferation. Taken together, the morphologic, immunophenotypic, and molecular findings led to a final diagnosis of DLBCL, non-germinal center B-cell (non-GCB) type.

## Discussion

This case illustrates a clinically important diagnostic pitfall: a markedly hypermetabolic mesenteric root mass may represent lymphoma even when preoperative imaging does not specifically suggest lymphoma. The practical significance is substantial because the management of DLBCL differs fundamentally from that of resectable intra-abdominal solid tumors. While surgery may be appropriate for selected solid tumors, DLBCL is primarily treated with systemic therapy once adequate diagnostic tissue has been obtained [5-7]. Conversely, insufficient tissue may lead to an incomplete diagnosis because immunohistochemistry, FISH, and selected molecular tests are often required to separate DLBCL from related aggressive B-cell neoplasms and from non-lymphoid tumors [1-4].

Diagnostic implications of very high FDG uptake in intra-abdominal tumors

The markedly elevated SUVmax in this case raised concern for an aggressive malignant process. However, very high FDG uptake is not specific for lymphoma. Aggressive B-cell lymphomas, including DLBCL, are typically FDG-avid, and higher FDG uptake has been associated with aggressive lymphoma biology, including higher-grade histology and proliferative activity, in several studies [8,11,12]. Nevertheless, similar metabolic intensity can also be observed in other intra-abdominal malignancies. GISTs may show FDG uptake that correlates with malignant potential and early treatment response, and soft tissue sarcomas may demonstrate a relationship between FDG uptake and tumor grade [13-15]. Poorly differentiated carcinomas, neuroendocrine carcinomas, melanoma, and metastatic tumors may also be highly FDG-avid [16].

At present, the available literature does not allow a reliable estimate of the proportion of lymphoma among intra-abdominal malignant tumors with SUVmax greater than 30. Most studies report standardized uptake value (SUV) distributions within specific tumor types rather than the pathological spectrum of abdominal tumors selected by an SUV threshold [11-16]. Therefore, an SUVmax of 35.5 in the present patient supported aggressive tumor biology but could not distinguish DLBCL from other high-grade intra-abdominal malignancies. This point is important because using SUV intensity alone to infer histology could bias the operative plan toward radical resection when tissue diagnosis should instead remain the priority.

The distinctive feature of this case was that none of the preoperative imaging interpretations specifically favored lymphoma. The lesion appeared as a dominant mesenteric root mass encased by small bowel loops and closely associated with mesenteric vessels, leading to a preoperative impression of an aggressive intra-abdominal solid tumor or metastatic disease. This diagnostic uncertainty was clinically important because management differs substantially between lymphoma and solid tumors. In such a scenario, intraoperative frozen-section analysis may provide critical real-time information and prevent irreversible, non-therapeutic extensive resection.

Why was lymphoma difficult to suspect preoperatively?

Several features may have contributed to the preoperative diagnostic difficulty. First, the mass was located at the mesenteric root and appeared to involve or encase adjacent bowel and vascular structures, a pattern that can be seen in aggressive solid tumors as well as lymphoma. Second, the lesion was interpreted as a dominant mass rather than a classic presentation of disseminated nodal lymphoma. Third, although mild FDG uptake was noted in several peripheral lymph node stations, these findings were not sufficiently specific to redirect the preoperative impression toward lymphoma. Finally, the extremely high SUVmax indicated aggressive disease but did not provide histologic specificity.

Although this is a single case, the findings suggest a cautious diagnostic approach to hypermetabolic intra-abdominal masses. Lymphoma may warrant consideration in the differential diagnosis even when the radiologic pattern is atypical, particularly when a planned operation might require extensive bowel resection or mesenteric vascular sacrifice.

Role of tissue diagnosis and frozen section

For suspected lymphoma, adequate tissue is essential not only to establish the diagnosis but also to perform immunohistochemistry, FISH, and molecular studies that guide classification and treatment [1-7]. Core needle biopsy is often preferred when feasible, because it may avoid surgery while providing tissue for ancillary studies. In this patient, however, the lesion location and proximity to bowel and mesenteric vessels made percutaneous biopsy unsafe after multidisciplinary review. Surgical exploration was therefore justified as a diagnostic procedure, but the operative objective needed to remain flexible until pathology clarified whether the lesion was a solid tumor or a hematolymphoid malignancy.

The key operative decision was not simply whether to remove the mass, but whether to proceed with extensive bowel and mesenteric vascular resection before a definitive diagnosis was available. Frozen-section analysis was decisive because it suggested a hematolymphoid malignancy during the operation. This information changed the surgical goal from radical resection to diagnostic tissue acquisition. By stopping the operation after adequate tissue sampling, the team avoided a potentially morbid procedure that would not have been the preferred initial treatment for DLBCL.

Surgical strategy after unexpected suspicion of lymphoma

The intraoperative decision to abandon extensive resection was consistent with the principle that surgery in DLBCL is generally reserved for diagnosis or for management of complications such as obstruction, perforation, hemorrhage, or selected localized residual disease after systemic therapy assessment [5-7,17-19]. The role of surgery in intestinal or mesenteric lymphoma remains context-dependent: emergency or complication-directed surgery may be necessary, whereas non-therapeutic radical resection before diagnostic confirmation can expose patients to avoidable morbidity [17-19]. In this case, there was no immediate indication for bowel or vascular resection once frozen section suggested lymphoma. Preserving bowel length and avoiding mesenteric vascular injury were therefore clinically appropriate.

If systemic therapy fails to control the disease, or if the patient later develops complications such as obstruction, perforation, bleeding, or a localized resectable residual lesion after hematologic evaluation, surgery may be reconsidered. This possibility should be framed as individualized salvage or complication-directed management rather than routine treatment for DLBCL.

Pathologic considerations

The final diagnosis was supported by strong B-cell marker expression, high proliferative activity, and molecular findings [1-4]. The tumor was CD20-positive and CD19-positive, with BCL6 and MUM1 expression and CD10 negativity, supporting classification as a non-germinal center B-cell type according to the pathology interpretation. The Ki-67 index of approximately 90% was consistent with highly proliferative aggressive lymphoma. BCL6 rearrangement was detected, while MYC and BCL2 rearrangements were absent; therefore, the available data did not support classification as high-grade B-cell lymphoma with MYC and BCL2 rearrangements under current classification principles [1,2]. MYC protein expression of approximately 40% should be distinguished from MYC gene rearrangement, which was negative.

Limitations

This report has several limitations. First, long-term follow-up and treatment response data were not available at the time of manuscript preparation. As a result, the clinical course after referral for systemic therapy could not be assessed. Second, this is a single case, and the findings should not be generalized to all hypermetabolic mesenteric root tumors. Third, although PET/CT showed a dominant mesenteric root mass, mild FDG uptake in other lymph node stations means that the case should be described cautiously as DLBCL presenting as a dominant mesenteric root mass rather than definitively as primary mesenteric DLBCL unless complete staging supports that designation.

## Conclusions

This case provides three practical lessons. First, a very high SUVmax in an intra-abdominal mass suggests aggressive tumor biology but is not histology-specific and cannot reliably distinguish DLBCL from high-grade solid malignancies. Second, when percutaneous biopsy of a mesenteric root mass is unsafe, surgical exploration may reasonably prioritize tissue diagnosis over radical resection in this clinical context. Third, intraoperative frozen-section analysis may be valuable when lymphoma is not suspected preoperatively, because it may help prevent unnecessary extensive bowel and mesenteric vascular resection and redirect management toward appropriate systemic therapy.

## Appendices

Supplementary Table A1

**Table 2:** Immunohistochemical (IHC) staining results (intensity/positivity). Intensity/positivity is reported as described in the immunohistochemical report (e.g., “+”, “++/+++”, and selected percentages).

Marker (IHC)	Result (as reported)	Comment/Notes
CD20	+++	Strong positivity
CD19	+++	Strong positivity; supports B-cell lineage
CD79a	+++	Strong positivity; supports B-cell lineage
CD21	+	Positive
BCL2	−	Negative
BCL6	+	Positive
CD10	10%+	Low-level positivity
CD23	Scant positive cells	Scant positivity
CD3	Positive in small T cells	Highlights background small T cells, not the atypical B-cell population
CD5	Positive in small T cells	Highlights background small T cells, not the atypical B-cell population
EBER	−	Negative
Ki-67 (MIB-1)	Approximately 90% positive	High proliferation index
MUM1/IRF4	+	Positive; supports non-germinal center B-cell phenotype in the appropriate context
CD30	−	Negative
CKpan	−	Negative
c-MYC	40%+	Positive in approximately 40% of tumor cells
p53 (DO7)	90%+++	Abnormally strong positivity in approximately 90% of tumor cells
p40	−	Negative
SYN	−	Negative
CgA	−	Negative
ALK (D5F3, Ventana)	−	Negative
ARID1α	+	Retained expression
BRG1	+	Retained expression
BRM	+	Retained expression
INI-1	+	Retained expression
MLH1	+	Retained expression
MSH2	+	Retained expression
MSH6	+	Retained expression
PMS2	+	Retained expression
CD56	−	Negative
CD43	−	Negative

Supplementary Table A2

**Table 3:** T and B cell receptor gene rearrangement analysis. Gene rearrangement analysis demonstrated clonal IGH rearrangement and concurrent clonal TCRB rearrangement. These findings were interpreted in conjunction with morphology, immunophenotype, and FISH results, which supported a diagnosis of diffuse large B-cell lymphoma. IGH: immunoglobulin heavy chain; TCRB: T-cell receptor beta; FISH: fluorescence in situ hybridization; BCR: B-cell receptor; TCR: T-cell receptor.

Gene type	Detection method	Target region	Product size range	Detection result	Clinical interpretation
B Lymphocyte	IGH Tube A	FR1-JH	310–360 bp	Positive (+)	Clonal BCR rearrangement detected
B Lymphocyte	IGH Tube B	FR2-JH	250–295 bp	Negative (−)	No rearrangement in this region
B Lymphocyte	IGH Tube C	FR3-JH	100–170 bp	Negative (−)	No rearrangement in this region
B Lymphocyte	IGK Tube A	VK-JK	120–160, 190–210, 260–300 bp	Negative (−)	No kappa light chain rearrangement
B Lymphocyte	IGK Tube B	VK-Kde+intron-Kde	210–250, 270–300, 350–390 bp	Negative (−)	No kappa light chain rearrangement
T Lymphocyte	TCRB Tube A	Vβ+Jβ	240–285 bp	Positive (+)	Clonal TCR beta rearrangement detected
T Lymphocyte	TCRB Tube B	Vβ+Jβ	240–285 bp	Negative (−)	No rearrangement in this primer set
T Lymphocyte	TCRB Tube C	Dβ+Jβ	170–210, 285–325 bp	Negative (−)	No delta-joining rearrangement
T Lymphocyte	TCRG-6FAM	Vg9+Jgl/Jg2, Vg10+Jg1/Jg2	159–207 bp	Negative (−)	No gamma rearrangement

Supplementary Table A3

**Table 4:** MYD88 gene mutation analysis. The absence of MYD88 mutation in this case does not exclude lymphoma but may guide subtype classification.

Gene	Exon	Detection method	Result	Interpretation
MYD88	Exon 5	Next-generation sequencing	Negative (−)	No mutation detected

Supplementary Table A4

**Table 5:** FISH analysis for chromosomal abnormalities and gene rearrangements. Both 11q23.3/11q24.3 deletions and IRF4 rearrangements are negative by established diagnostic thresholds. IRF4 translocations are typically associated with primary cutaneous lymphomas; the absence of this finding is consistent with the diagnosis of a systemic lymphoproliferative disorder. FISH: fluorescence in situ hybridization.

Target	Probe	Cells analyzed	Abnormal cells	Abnormal percentage	Gene status	FISH result
11q23.3/11q24.3	Deletion probe	100	10	10%	4% gain at 11q23.3; 6% loss at 11q24.3	Negative (−)
IRF4(6p25)	Breakpoint probe	100	0	0%	No signal separation	Negative (−)
